# Extra-axial cerebrospinal fluid volumes from 6 to 24 months of age are associated with poorer executive function at school-age in children with and without autism

**DOI:** 10.1186/s11689-025-09671-z

**Published:** 2026-01-16

**Authors:** Yichi Zhang, Joshua Rutsohn, Sun Hyung Kim, Juhi Pandey, Robert T. Schultz, Lonnie Zwaigenbaum, Catherine Burrows, Stephen R. Dager, Tanya St. John, Annette M. Estes, Robert C. McKinstry, Natasha Marrus, John R. Pruett, Martin Styner, Heather C. Hazlett, Joseph Piven, Mark D. Shen, Dea Garic

**Affiliations:** 1 Carolina Institute for Developmental Disabilities, University of North Carolina School of Medicine, Chapel Hill, NC 27599 USA; 2 Department of Psychiatry, University of North Carolina School of Medicine, Chapel Hill, NC 27599 USA; 3 Center for Autism Research, Children’s Hospital of Philadelphia, Philadelphia, PA 19104 USA; 4 Department of Pediatrics, University of Alberta, Edmonton, AB T6G 2R3 Canada; 5 Department of Pediatrics, Medical School, University of Minnesota, Minneapolis, MN 55455 USA; 6 Department of Radiology, University of Washington, Seattle, WA 53792 USA; 7 Department of Speech and Hearing Sciences, University of Washington, Seattle, WA 98105 USA; 8 Mallinckrodt Institute of Radiology, Washington University in Saint Louis School of Medicine, St. Louis, MO 63110 USA; 9 Department of Psychiatry, Washington University in Saint Louis School of Medicine, St. Louis, MO 63110 USA; 10 Department of Computer Science, University of North Carolina at Chapel Hill, Chapel Hill, NC 27599 USA

**Keywords:** Extra-axial cerebrospinal fluid, MRI, Executive function, Autism

## Abstract

**Background:**

Abnormally increased extra-axial cerebrospinal fluid (EA-CSF) volume is present as early as 6 months in infants later diagnosed with autism and is associated with symptom severity at the age of diagnosis, but it is unknown whether early EA-CSF enlargement has long-term impacts on other clinical domains. Executive function (EF) deficits are frequently observed in children with autism and are linked with worse academic outcomes, higher anxiety, and lower adaptive functioning. The current study examines the association between EA-CSF volume at infancy and EF at school age in a longitudinally phenotyped cohort of children with either high (HL) or low (LL) familial likelihood for autism.

**Methods:**

In this prospective study, 239 infants underwent MRI scans during natural sleep at 6, 12, and 24 months of age. HL was defined as having an older sibling with autism. The sample was divided into three diagnostic groups: HL infants who were diagnosed with autism at 24 months (HL+, *n* = 34), HL infants not diagnosed with autism (HL-, *n* = 131), and LL infants without autism (LL-, *n* = 74). Two parent-rating scales of EF were collected at the school-age follow-up (*M*_age_= 10.4 years ± 1.34): the Behavior Rating Inventory of Executive Function (BRIEF) and Conners Parent Short Form. ANOVA was used to test for diagnostic group differences in EF. Longitudinal mixed-effects models utilized EA-CSF volumes in infancy to predict EF at school-age, while controlling for IQ, sex, total cerebral volume, and diagnostic group.

**Results:**

Consistent with previous literature, HL + participants had greater EF deficits than both HL- and LL- on the BRIEF (*F* = 18.46, *p* < .0001) and greater EF deficits than LL- on the Conners (*F* = 8.55, *p* < .001). Further, higher EA-CSF volumes during infancy were associated with poorer executive function eight years later on both BRIEF (*ß*=0.18, *p* < .001) and Conners (*ß*=0.18, *p* < .001). This association between EA-CSF and executive function was observed across familial likelihood and diagnostic groups.

**Conclusions:**

Our study indicates that elevations in EA-CSF volumes during infancy, even in those who do not have autism, have long-term associations beyond autism symptomatology. These findings underscore the potential link between infant CSF physiology and future behavioral domains such as executive function at school age.

**Supplementary Information:**

The online version contains supplementary material available at 10.1186/s11689-025-09671-z.

## Introduction

Autism is a spectrum of neurodevelopmental conditions defined by social and communication difficulties as well as repetitive, stereotypic behaviors [1], with a US prevalence rate of 2.7% [2]. Cerebrospinal fluid (CSF) plays a crucial role in early neurodevelopment by circulating essential growth factors and facilitating waste clearance [3]. Extra-axial cerebrospinal fluid (EA-CSF) refers to CSF that flows in the subarachnoid space surrounding the brain [4]. Elevated EA-CSF volumes from 6 to 36 months of age have been associated with autism diagnosis before the onset of symptoms [5–7]. This finding has been independently replicated in a community ascertained sample in Israel [8], suggesting that EA-CSF volume can serve as a potential stratification biomarker to identify a subgroup of children with autism with a specific etiology. EA-CSF volume at 6 months of age has been correlated with motor delays at 12 months and greater autism symptomology at diagnosis [5, 9, 10]. Although the association between EA-CSF volumes and autism diagnosis and severity has been documented during infancy, the long-term impact of EA-CSF accumulation during early brain development is largely unexplored.

Autism commonly co-occurs with other neurodevelopmental conditions, as there is a shared genetic basis for the presentation of symptomology [11]. Younger siblings of autistic children are more likely to develop autism themselves [12]. Even siblings of autistic children who do not develop autism are at a higher likelihood of developing other developmental disabilities, such as attention-deficit/hyperactivity disorder (ADHD), that share overlapping behavioral symptoms with autism, like executive function deficits [13, 14]. Given that family history of autism suggests a broader susceptibility for neurodevelopmental concerns, a transdiagnostic approach is needed to encompass the range of difficulties faced by this population and identify supports that may be needed even in the absence of clinical diagnoses.

Executive function difficulties span across neurodevelopmental conditions in clinical and subclinical samples [15, 16]. Executive functions (EFs) refer to skills that enable individuals to manage and regulate their cognitive processes in order to achieve goals [17]. For individuals with autism, EF difficulties have been associated with worse academic outcomes, higher anxiety symptoms, and lower adaptive functioning [18–20]. In a large population sample, impaired EF in school-age children was associated with more traits of autism and ADHD [21]. A latent profile analysis of seven EF domains in childhood did not find specific EF profiles to align with diagnosis, but rather suggested the presence of EF deficits across autism and ADHD [22]. Given that EF deficits are strongly associated with a range of negative outcomes across neurodevelopmental conditions, EF presents great utility as a transdiagnostic marker of neurodevelopment [23–26].

Measuring EF is difficult due to the complexity of skill and situational context being assessed. Behavioral tasks and questionnaires are both used to measure EF [27]. Behavioral performance tasks are designed to measure abilities in a controlled test environment, while parent-report questionnaires focus on outcomes resulting from applying EF in daily life. Both types of measures provide valuable information, though they assess different aspects of EF. In populations with autism, differences can be exacerbated as individuals may perform better in structured rather than unstructured environments [28]. Therefore, parent-report questionnaires may represent higher ecological validity and help characterize the clinical relevance of EF in populations with autism [21, 29]. Further, synthesizing EF research is challenged by the differing cognitive abilities and occurrences of intellectual disability in children with autism [29, 30]. Intelligence and IQ have been linked with EF in school-age children, making it necessary to parse out the effects of EF from the influence of IQ [31–33].

The goal of our current study is to examine whether infant EA-CSF volumes are associated with parent-reported EF measures approximately 8 years later, across familial likelihood and diagnosis for autism. Specifically, our aims are (1) to determine whether EF difficulties are stratified by familial likelihood and autism diagnosis, (2) to examine longitudinal associations of infant EA-CSF volumes and school-age EF, and (3) to explore whether group brain-behavior interactions drive these associations.

## Method

### Participants

The 239 participants (147 male) included in this study are from the Infant Brain Imaging Study (IBIS), a prospective longitudinal study that examines early brain and behavior development in autism. Infants are classified as either high familial likelihood (HL) for autism by virtue of having an older sibling diagnosed with autism or low familial likelihood (LL) if they have no family history of autism. Families were recruited at four sites in North Carolina, Missouri, Pennsylvania, and Washington by IBIS-affiliated universities. Participants were classified into three groups depending on their familial likelihood for autism and their diagnosis at 24 months of age. The HL + group (*n* = 34) consisted of participants who had a sibling with autism and received a diagnosis themselves; HL- (*n* = 131) consisted of participants who had an older sibling with autism but did not receive an autism diagnosis themselves; and LL- (*n* = 74) group served as a control with no family history of autism and no autism diagnosis. The inclusion and exclusion criteria for this sample have been detailed by Hazlett et al. (2012) [34]. For the current analysis, participants must have had at least one MRI scan between 6 and 24 months and returned for their school-age assessment (*M*_age_= 10.4 years ± 1.34), where the parent measures were collected.

### Procedures

Study procedures were approved by the Institutional Review Board of each site where data collection took place. Once enrolled in IBIS, participants underwent brain imaging and behavioral assessment at 6, 12, and 24 months of age. The clinical diagnosis for participants was determined by a comprehensive clinical evaluation at the 24-month visit according to Diagnostic and Statistical Manual-IV (DSM-IV) guidelines, using the Social Communication Questionnaire [37] and the Autism Diagnostic Interview–Revised [36]. A subset of participants returned approximately eight years later for a school-age visit, during which measures related to developmental level and symptoms were collected through behavioral assessments and parent questionnaires. Data collection and access were centralized from each site on the LORIS data management platform [37].

### MRI acquisition

MRI scans were collected during natural sleep at the 6-, 12-, and 24-month timepoints, with the number of scans displayed in Table 1. T1- and T2-weighted images (1 mm³ voxels) were captured using a 3T Tim Trio scanner with a 12-channel head coil at each of the four data collection sites [38]. Scans were reviewed by a pediatric radiologist for incidental radiological findings. Then, scans underwent quality assessment with a pass/fail designation from blind raters. Total cerebral volume (TCV) was obtained from a previously published protocol [7]. Quantification of EA-CSF volume was generated by an automated multi-atlas segmentation method developed by our team [5].

### Executive function rating measures

#### Behavior Rating Inventory of Executive Function, 2nd edition (BRIEF)

The BRIEF is a parent questionnaire that assesses the day-to-day executive functioning of children aged 5–18 [39]. For each of the 63 items on the questionnaire, parents are asked to rate each statement of their child’s behaviors from the past six months as “Never”, “Sometimes”, or “Often”. The results from this questionnaire include three index scores: Behavioral Regulation, Emotional Regulation Index, and Cognitive Regulation Index, as well as an overall Global Executive Composite (GEC). The GEC T-score is normalized for age and sex and is used in our study to represent executive function, with higher scores indicating greater difficulties with executive function. The internal consistency of the BRIEF was excellent in our sample with α = 0.96.

#### Conners-3 parent short form (Conners-P)

The Conners-P is a short parent questionnaire designed to evaluate ADHD symptomology in children aged 6–18 [40]. The questionnaire includes 31items rated on a four-point Likert scale, ranging from zero (Never) to three (Very Frequently). Six sub-scores were generated for the content scales: Inattention, Hyperactivity/Impulsivity, Learning Problems, Executive Functioning, Defiance/Aggression, and Peer Relations. The Executive Functioning T-score, which controls for age and sex, is used in our analysis, where higher scores represent more reported executive function difficulties. The internal consistency of Conners-P was also excellent in our sample with α = 0.95.

### School-age IQ measure from the DAS-II

The Differential Ability Scales Second Edition (DAS-II) for school-age children (ages 7–17) is a cognitive ability battery comprising 20 individually administered subtests [41]. The DAS-II generates an overall composite score, known as the general conceptual ability (GCA), with a mean of 100 and a standard deviation of 15. The GCA score is calculated based on six core subtests: verbal comprehension, naming vocabulary, picture similarities, matrices, pattern construction, and copying. The GCA score has been reported to have high correlations with overall composite scores of other cognitive batteries, such as the Stanford–Binet, Woodcock–Johnson, and Wechsler Intelligence Scale for Children [42]. The reliability and validity of the DAS-II have been documented in independent studies [43].

### Data analysis

Analysis of variance (ANOVA) and post-hoc analysis using Tukey’s method were employed to examine diagnostic group differences in study measures among the LL-, HL-, and HL + groups. Longitudinal mixed-effects modeling was employed to investigate the relationship between infant EA-CSF volumes at 6, 12, and 24 months of age and school-age EF measures from the BRIEF and the Conners-P. All models contained fixed effects of mean EA-CSF volume and TCV at each available time point, as well as sex, diagnostic group, and school-age GCA score. For each EF measure, we tested the model with and without an interaction term to examine the brain-behavior interaction between EA-CSF and each diagnostic group. For the random effects in each model, random slopes and intercepts were created for EA-CSF volumes nested within each of the three visits and diagnostic groups. A continuous autoregressive covariance structure was chosen in our model to account for the individual continuity of EA-CSF volumes during infancy and the unbalanced study design. The data presented are the fixed main effect of each individual’s mean EA-CSF across infant visits (with the random effect accounting for the group x visit interaction). In exploratory analyses, we included *post-hoc* linear mixed-effects models to test associations between *trajectories* of EA-CSF volume (i.e., an individual’s EA-CSF change over time) between 6 and 24 months of age and later EF outcomes. All models were run in R version 4.4.2 using the nlme package [44, 45].

## Results

### Descriptive statistics by diagnostic group

Table 1 displays the descriptive statistics by diagnostic group for age, sex, and all measures used in our models. A chi-square test detected group differences in sex distribution between the diagnostic groups (χ²(2, *N* = 239) = 13.23, *p* = .001) with pairwise Bonferroni comparisons indicating that the HL + group (88% male) differed significantly from both the HL − group (54% male; *p* = .002) and LL − group (62% male; *p* = .034).

**Table 1:** Descriptive statistics by diagnostic group for all study variables

Characteristic	HL+	HL-	LL-	F or X^2^	*p*	post-hoc contrasts
Diagnostic Group, No.	34	131	74			
Sex, Male No. (%)	30 (88%)	71 (54.2%)	46 (62.2%)	X2 = 13.23	< 0.01	LL- < HL+, HL- < HL+
Infant Ages, month (SD)						
6-month MRI	6.46 (0.54)	6.63 (0.64)	6.72 (0.73)	F = 1.67	0.19	N/A
12-month MRI	12.79 (0.76)	12.6 (0.62)	12.70 (0.67)	F = 1.27	0.28	N/A
24-month MRI	24.66 (0.63)	24.66 (0.83)	24.67 (0.74)	F = 0.01	0.99	N/A
Infant MRI Scans						
6-month, No.	24	89	61			N/A
EA-CSF Volume (cm^3^)	85.14 (35.50)	71.98 (21.80)	75.38 (20.10)	F = 2.96	0.055	N/A
TCV Volume (cm^3^)	694.21 (59.26)	672.98 (60.26)	694.00 (57.90)	F = 2.8	0.064	N/A
12-month, No.	25	112	60			N/A
EA-CSF Volume (cm^3^)	76.19 (28.38)	70.69 (19.10)	73.12 (19.52)	F = 0.83	0.44	N/A
TCV Volume (cm^3^)	858.20 (73.58)	802.66 (71.93)	816.545 (66.24)	F = 6.64	< 0.01	LL- < HL+, HL- < HL+
24-month, No.	24	101	47			N/A
EA-CSF Volume (cm^3^)	73.03 (14.78)	69.85 (14.82)	70.68 (13.42)	F = 0.47	0.62	N/A
TCV Volume (cm^3^)	983.18 (99.07)	936.67 (85.03)	952.50 (86.60)	F = 3.26	< 0.05	HL- < HL+
School-age Visit						
Age, months (SD)	124.29 (13.76)	127.36 (16.87)	121.25 (15.44)	F = 3.22	< 0.05	LL- < HL-
Conners-P, No.	32	129	72			N/A
Conners-P EF T-Score	63.34 (14.28)	58.68 (15.34)	51.9 (12.33)	F = 8.55	< 0.001	LL- < HL-, LL- < HL+
BRIEF-P, No.	25	101	58			N/A
BRIEF-P GEC T-Score	64.76 (12.50)	51.53 (11.30)	49.1 (9.98)	F = 18.46	< 0.0001	LL- < HL+, HL- < HL+
DAS, No.	33	123	74			N/A
DAS GCA Score	98.18 (22.1)	109.61 (14.47)	115.53 (15.54)	F = 13.29	< 0.0001	all groups

*HL* + high familial likelihood for autism and autism diagnosis, *HL*-  high familial likelihood for autism and no autism diagnosis, *LL*-  low familial likelihood for autism and no autism diagnosis, *TCV*  Total Cerebrum Volume, *EA-CSF*  Extra-Axial Cerebrospinal Fluid

### Children with autism had significantly greater executive function difficulties

One-way ANOVAs revealed significant differences among diagnostic groups on both executive function measures and the school-age IQ score. A one-way ANOVA detected differences for EF difficulties on the BRIEF GEC (*F*(2, 181) = 18.46, *p* < .0001, η² = 0.17). Post hoc comparisons indicated that the HL + group (M = 64.76, SD = 12.5) had significantly higher execution function difficulties than the HL- group (M = 51.53, SD = 11.3; *p* < .0001) and LL- group (M = 49.1, SD = 9.98; *p* < .0001). Group differences were also detected for the Conners EF score (*F*(2, 230) = 8.54, *p* < .001, η² = 0.07). Post hoc analysis determined that the HL + group (M = 63.34 SD = 14.28) had greater executive function difficulties than the LL- group (M = 51.9, SD = 12.33, *p* < .001), but no statistical difference was found against the HL- group (M = 58.68, SD = 15.34; *p* = .23). The HL- group also had greater EF difficulties than the LL- group (*p* < .01). For the school-age IQ measure, the DAS GCA score differed significantly by group (*F*(2, 227) = 13.29, *p* < .0001, η² = 0.10). Post hoc comparisons indicated that the HL + group (M = 98.18, SD = 22.1) had lower IQ scores than the HL- group (M = 109.61, SD = 14.47; *p* < .01) and LL- group (M = 115.53, SD = 15.54; *p* < .0001). The HL- group also had lower IQ scores than the LL- group (*p* < .05).

### Infant EA-CSF volume is linked to Parent-reported executive function

**Fig. 1:**
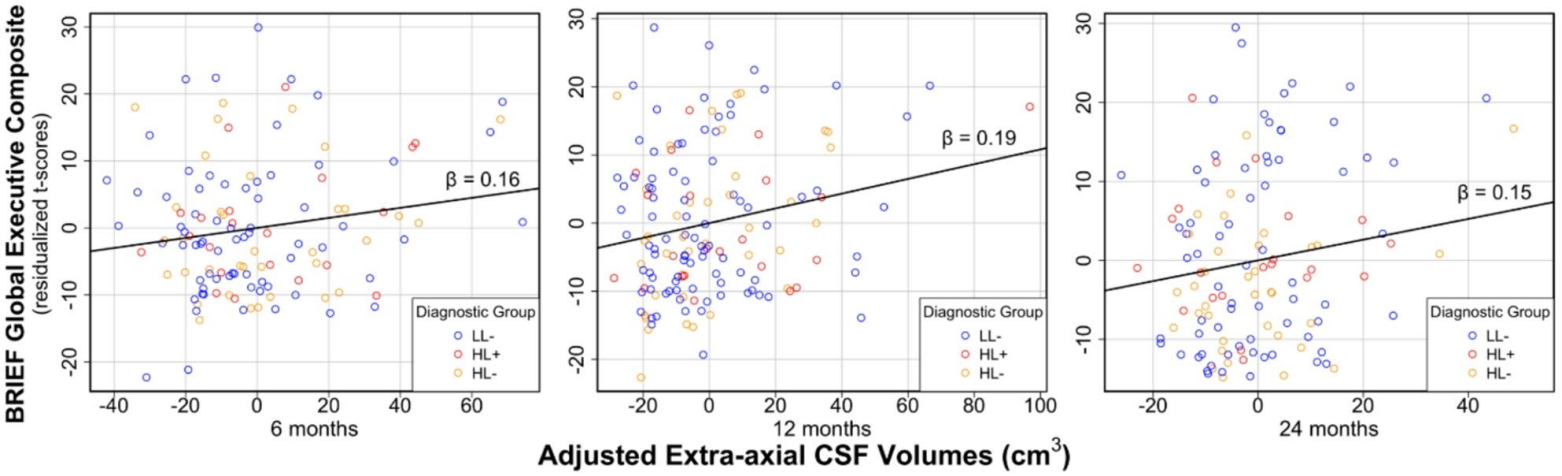
Infant EA-CSF volumes are associated with poorer school-age executive function on the BRIEF GEC. Partial regression plots show that higher EA-CSF volumes at 6, 12, and 24 months of age are significantly associated with higher scores (i.e., greater executive dysfunction) on the BRIEF Global Executive Composite (*β* = 0.18; *p* < .001). Cross-sectional beta values for each time point (β = 0.16, 0.19, and 0.15, respectively) are displayed above the trend line. Analyses covary for school-age IQ, diagnostic group, sex, and total cerebrum volume

EA-CSF volume from 6 to 24 months of age was a positive predictor for EF difficulties at school-age on the BRIEF, while covarying for school-age IQ, diagnostic group, sex, and TCV (Table 2; Fig. 1; *β* = 0.18; *SE* = 0.05; 95% *CI* [0.09, 0.27]; *t* = 3.81; *p* < .001). Examining covariates, the DAS GCA score was statistically significant, with higher GCA scores associated with lower EF difficulties (*β* = -0.20; *SE* = 0.05; 95% *CI* [-0.30, -0.11]; *t* = -4.14; *p* < .0001). The main effect of diagnostic group was also significant in the model (*F* = 23.83; *p* < .01), indicating that children in the HL- group (*β* = -0.77; *SE* = 0.16; 95% *CI* [-1.22, -0.32]; *t* = -4.75; *p* = .009) and LL- group (*β* = -0.93; *SE* = 0.17; 95% *CI* [-1.42, -0.45]; *t* = -5.38; *p* = .006) had lower EF difficulties as indexed by BRIEF-GEC at school-age compared to children in the HL + group. Sex and TCV were nonsignificant in the BRIEF model.

**Table 2:** Longitudinal model results for BRIEF-GEC predicted by EA-CSF and covariates

Predictor variables: Main effects	df (model)	df (residual)	F	*p*		
Intercept	1	375	8291.73	< 0.0001		
EA-CSF Volume	1	375	22.25	< 0.0001		
Total Cerebrum Volume	1	375	0.51	0.475		
DAS-II GCA Score	1	375	17.12	< 0.0001		
Sex	1	375	0.02	0.89		
Diagnostic Group	2	4	23.83	< 0.01		
Predictor variables: Parameter estimates	*df*	*β*	*SE*	95% *CI*	*t*	*p*
Intercept	375	0.74	0.16	[0.41, 1.06]	4.48	< 0.001
EA-CSF Volume	375	0.18	0.05	[0.09, 0.27]	3.81	< 0.001
Total Cerebrum Volume	375	0.03	0.05	[-0.07, 0.13]	0.50	0.618
DAS-II GCA Score	375	-0.20	0.05	[-0.30, -0.11]	-4.14	< 0.001
Sex: male	375	-0.02	0.10	[-0.21, 0.18]	-0.17	0.862
Diagnostic Group: LL-	4	-0.93	0.17	[-1.42, -0.45]	-5.38	0.006
Diagnostic Group: HL-	4	-0.77	0.16	[-1.22, -0.32]	-4.75	0.009

EA-CSF volume refers to the fixed effect for the overall mean of EA-CSF volumes in the model. Diagnostic Group variables are referenced against HL+. *HL* +  high familial likelihood for autism and autism diagnosis, *HL*- high familial likelihood for autism and no autism diagnosis, LL- low familial likelihood for autism and no autism diagnosis, *df*  degrees of freedom, *SE*  standard error, *CI*  confidence interval

**Fig. 2:**
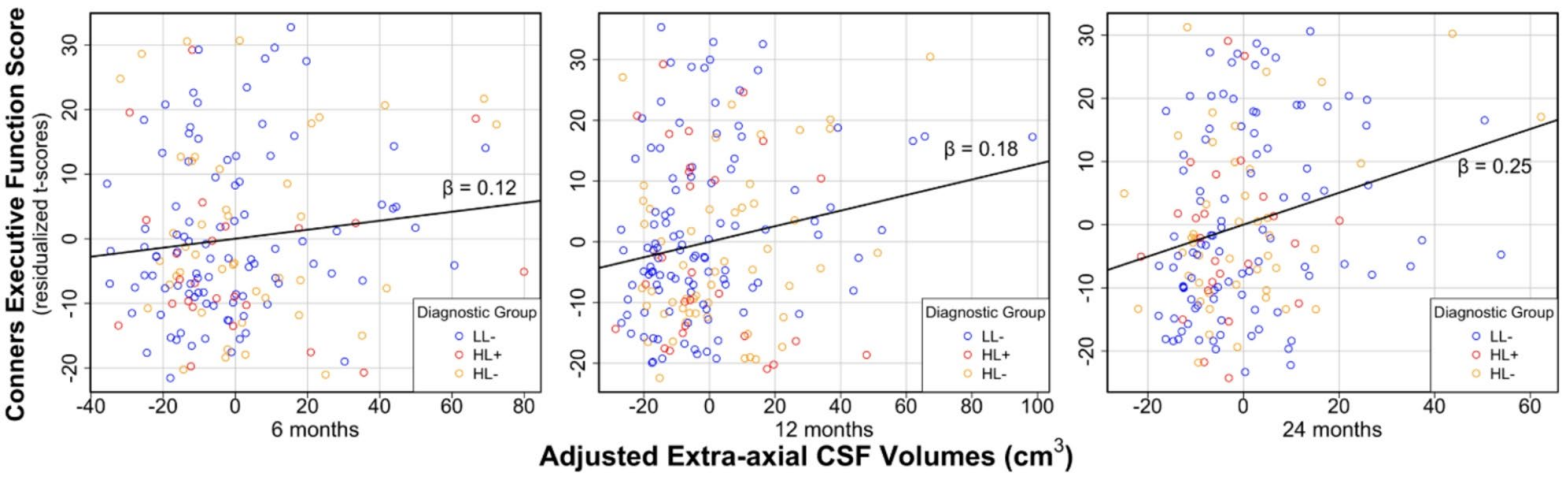
Infant EA-CSF volumes are associated with poorer school-age executive function on the Conners EF. Partial regression plots show that higher EA-CSF volumes at 6, 12, and 24 months of age are significantly associated with higher scores (i.e., greater executive dysfunction) on the Conners Executive Function composite (*β* = 0.18; *p* < .001). Cross-sectional beta values for each time point (β = 0.12, 0.18, and 0.25, respectively) are displayed above the trend line. Analyses covary for school-age IQ, diagnostic group, sex, and total cerebrum volume

The second model using the Conners EF score (see Fig. 2) showed similar results to the BRIEF model. There was a significant main effect of greater EA-CSF volumes from 6 to 24 months of age on higher EF difficulties, as measured by the Conners (Table 3; Fig. 2; *β* = 0.18; *SE* = 0.05; 95% *CI* [0.08, 0.28]; *t* = 3.63; *p* < .001). Like the BRIEF model, the DAS GCA score held a negative association with Conners EF difficulties (*β* = -0.18; *SE* = 0.05; 95% *CI* [-0.28, -0.08]; *t* = -3.59; *p* = .0004), indicating higher GCA is linked to lower EF difficulties. The main effect of diagnostic group was significant in the model (*F* = 13.47; *p* = .02), with children in the LL- group scoring lower on EF difficulties on the Conners compared to the HL + group (*β* = -0.55; *SE* = 0.17; 95% *CI* [-1.01, -0.08]; *t* = -3.25; *p* = .031). Additionally, sex was a significant covariate in the Conners model with males scoring lower on EF difficulties compared with females (*β* = -0.22; *SE* = 0.10; 95% *CI* [-0.43, -0.02]; *t* = -2.14; *p* = .033). TCV was not a statistically significant predictor in the model. While mean EA-CSF across 6–24 months was significantly associated with later EF outcomes, exploratory *post-hoc* analyses showed that *individual change in EA-CSF over time* was not significantly associated with later EF outcomes (*p* = .14 and 0.20 for BRIEF and Conners, respectively), indicating that infant EA-CSF and EF outcomes were associated regardless of an individual’s EA-CSF change over time. Tables 2 and 3 display results from the two longitudinal mixed models built to examine the effects of EA-CSF volumes during infancy on the BRIEF and Conners, respectively.

**Table 3:** Longitudinal model results for Conners-EF predicted by EA-CSF and covariates

Predictor variables: Main effects	df (model)		df (residual)		F	*p*		
Intercept		1	375	7091.00		< 0.0001		
EA-CSF Volume		1	375	12.09		< 0.001		
Total Cerebrum Volume		1	375	0.22		0.638		
DAS-II GCA Score		1	375	12.91		< 0.001		
Sex		1	375	3.72		0.054		
Diagnostic Group		2	4	13.47		0.017		
Predictor variables: Parameter estimates		*df*	*β*	*SE*		95% *CI*	*t*	*p*
Intercept		375	0.40	0.16		[0.08, 0.72]	2.48	0.014
EA-CSF Volume		375	0.18	0.05		[0.08, 0.28]	3.63	< 0.001
Total Cerebrum Volume		375	0.01	0.05		[-0.09, 0.11]	0.21	0.833
DAS-II GCA Score		375	-0.18	0.05		[-0.28, -0.08]	-3.59	< 0.001
Sex: male		375	-0.22	0.10		[-0.43, -0.02]	-2.14	0.033
Diagnostic Group: LL-		4	-0.55	0.17		[-1.01, -0.08]	-3.25	0.031
Diagnostic Group: HL-		4	-0.16	0.16		[-0.60, 0.28]	-1.02	0.366

EA-CSF volume refers to the fixed effect for the overall mean of EA-CSF volumes in the model. Diagnostic Group variables are referenced against HL+. *HL* +  high familial likelihood for autism and autism diagnosis, *HL*- high familial likelihood for autism and no autism diagnosis, *LL*- low familial likelihood for autism and no autism diagnosis. *df*  degrees of freedom, *SE*  standard error, *CI*  confidence interval

### Examining brain-behavior interaction by diagnostic group

For our third aim, we examined whether the effects of EA-CSF volume on EF were specific to a certain diagnostic group. We tested models with an additional interaction term between EA-CSF and diagnostic group in both EF models to examine whether EA-CSF held as a main effect across diagnostic groups, or whether the effect of EA-CSF volume was being driven by certain groups and not others. In both the BRIEF and Conners model, the interaction term was not a statistically significant predictor. In the BRIEF model, EA-CSF volume remained significant (*β* = 0.26; SE = 0.09; 95% CI [0.09, 0.43]; *t* = 3.03; *df* = 373; *p* = .003). For the Conners model, the EA-CSF volume also remained significant (*β* = 0.19; *SE* = 0.09; 95% *CI* [0.01, 0.36]; *t* = 2.05; *df* = 373; *p* = .041). These results show that the main effect of EA-CSF volume in infancy on parent-reported school-age executive function is not specific to familial likelihood or autism diagnosis but is observed across diagnostic groups.

## Discussion

Our study is the first to highlight the association between elevated infant EA-CSF volumes and developmental outcomes beyond early childhood. The main aim of our study was to examine the effects of EA-CSF volumes during infancy on executive function at school age, approximately eight years later, in a longitudinal sample of children with and without a higher familial likelihood for autism.

As expected, children with autism had greater executive function difficulties at school-age than both the HL- and LL- groups. Regardless of familial likelihood for autism and diagnostic status, EA-CSF volume during infancy remained a significant predictor for parent-reported EF at school-age. Similar to previous reports [7, 8, 46, 47], we observed significant group differences in total brain volume at 12–24 months of age, but our findings suggest that overall larger brain size was not specifically associated with executive function difficulties. EA-CSF volume, above and beyond what can be explained by diagnostic group and global differences in brain size, was associated with EF in our longitudinal models.

The literature on EA-CSF volumes has mainly focused on the early period of infancy through 48 months [6]. This observed phenomenon of elevated EA-CSF volumes in infancy is thought to resolve within the first few years of life. In a sample with ages 5 through 21, researchers did not find differences in EA-CSF volumes between groups for those with and without an autism diagnosis, suggesting that this biomarker may only be present in the first few years of life [48]. Understanding the link between EA-CSF volumes and clinically relevant EF outcomes requires investigating early etiologies of the CSF circulation.

Other transient early neural biomarkers have been reported in autism, with amygdala volumes being one example [49–52]. These markers of atypical brain development share the early time period of infancy and early childhood, highlighting how the first few years of life can have a lasting impact on long-term behavioral and clinical outcomes. This emphasizes a potentially crucial sensitive period in development, in which intervention and treatment can have the greatest clinical impact.

To examine EF outcomes at school age, we utilized composite measures from the parent-report BRIEF and Conners. We observed convergence across measures, with higher EA-CSF volumes during the first two years of life being negatively associated with both BRIEF GEC and Conners EF scores at school age. Broadly speaking, while both measures are widely used to assess EF, BRIEF focuses on EF ability, whereas Conners has a greater focus on EF deficits. In our study, the primary difference we observed between the two EF measures was that the BRIEF GEC was more sensitive to diagnostic group differences. Specifically, BRIEF GEC was able to detect differences between all three groups, with HL + scoring significantly higher than both HL- and LL-, while Conners EF was only able to detect significant differences between HL + and LL-. This could be helpful for future studies examining EF differences among children with higher familial likelihood for autism.

The findings of our study suggest that elevations in EA-CSF volume during the first two years of life could potentially be a risk factor for later executive dysfunction. There is growing evidence that early targeted intervention of EF can promote long-term improvements [53]. For example, a randomized clinical trial found that a child’s participation in tailored, family-centered training programs at the age of two was associated with greater inhibitory control by middle childhood, and subsequently lower externalizing behavior by adolescence [54]. Examining EA-CSF volume during infancy can potentially help identify which young children could benefit the most from early-life EF intervention.

Finally, our findings suggest that elevations in EA-CSF volume during infancy can have long-term impacts on EF outcomes for all children, regardless of diagnosis. While infants who go on to develop autism may have a greater chance of having elevated EA-CSF volumes [5, 7], we observed that greater EA-CSF, even in children who do not go on to develop autism, is potentially associated with long-term neurodevelopmental outcomes. Increased EA-CSF volume is thought to result from impaired CSF circulation, a vital process for distributing growth factors [55, 56] and clearing waste products in the brain [57]. Diminished CSF circulation may have brain-wide impacts during early neurodevelopment and could be an underlying mechanism contributing to long-term behavioral outcomes. Therefore, these findings provide further support for the notion that elevated EA-CSF volume, also referred to as benign extra-axial fluid of infancy or benign external hydrocephalus [58], may not be entirely benign and warrants further examination during critical early-life brain development.

### Strengths

Our current study has a number of strengths, including a large sample size of children followed for over 10 years, starting at 6 months of age to over 10 years old, a prospective cohort design, and an examination of two widely accepted EF measures across diagnostic categories. As part of a longstanding research network on brain development and autism, we had unique access to a large sample of infant brain images and follow-up behavioral data at school age. Consequently, we were able to establish a prospective relationship between EA-CSF volumes and executive functioning, while controlling for possible confounding factors of IQ. While IQ is a significant covariate for EF, EA-CSF volumes at infancy were significantly associated with school-age EF above and beyond the impact of IQ. The diagnostic grouping allows us to examine the stratification of EF by familial likelihood for autism, as well as autism diagnosis. In this way, we documented EF difficulties for groups with a higher genetic propensity for neurodevelopmental concerns. Lastly, we utilized two widely accepted, general EF measures, both of which yielded similar results and demonstrated links to infant EA-CSF, thereby demonstrating robustness and consistency in our findings.

### Limitations and future directions

Our study has several limitations that can be addressed in future studies, including the use of a broader range of EF measures, the underrepresentation of females in the autism group, and consideration of other factors that can impact EF outcomes. We only used composite measures from parent-rating scales to capture school-age EF. Given the heterogeneity and range of EF skills, more work is needed to parse the different subconstructs in EF and examine unique EA-CSF brain-behavior relationships. In addition to parent-rating scales, other informants (e.g., teachers) can be involved to create a more comprehensive view of the child’s EF across various environments and interpersonal relationships. Direct assessment of EF should also be examined, as it may capture different aspects of EF missed in parent-rating scales. In considering our sample, the HL + group is skewed heavily towards males (88%), which possibly limits the conclusions we can make about EA-CSF and EF in females with autism. Lastly, it is important to acknowledge that EF challenges likely arise from a multitude of factors, including other neurobiological characteristics (e.g., frontostriatal circuits) [59, 60] and physical health factors (e.g., sleep quality and duration) [61, 62]. EA-CSF volume is likely not a sole predictor of future EF outcomes but could be indicative of impaired CSF clearance that can widely impact early brain development and subsequent long-term EF. Future studies should examine the EA-CSF in conjunction with neural pathway development and sleep patterns, which are likely interlinked [6, 63].

## Conclusion

In conclusion, we found that elevated infant EA-CSF volumes are a significant predictor of school-age executive function outcomes, independent of overall brain size, familial likelihood for autism, and diagnostic status. By leveraging a large, prospective cohort, we established robust longitudinal links between early brain biomarkers and school-age EF while accounting for school-age IQ. These findings highlight EA-CSF as a potential early marker of transdiagnostic executive function difficulties; however, further research is needed to clarify the behavioral mechanisms and expand EF measurements beyond parent-report. Identifying infants with elevated EA-CSF provides an opportunity to detect those at risk for executive function difficulties and deliver targeted interventions during key developmental windows [53].

## Supplementary Information

Supplementary Material 1.

## Data Availability

The datasets utilized and analyzed in the current study can be made available by the corresponding author upon reasonable request, and subject to IRB approval. Data associated with ASD participants is included in the National Database for Autism Research (NDAR).

## Abbreviations

ADHD: Attention-Deficit/Hyperactivity Disorder
ANOVA: Analysis of Variance
Aβ: Amyloid Beta
BRIEF: Behavior Rating Inventory of Executive Function
Conners-P: Conners-3 Parent Short Form
CSF: Cerebrospinal Fluid
DAS-II: Differential Ability Scales, Second Edition
DSM-IV: Diagnostic and Statistical Manual of Mental Disorders, Fourth Edition
EA-CSF: Extra-Axial Cerebrospinal Fluid
EF: Executive Function
GCA: General Conceptual Ability
GEC: Global Executive Composite
HL: High Familial Likelihood for Autism
HL-: High Familial Likelihood for Autism, No Autism Diagnosis at 24 Month Visit
HL+: High Familial Likelihood for Autism, Autism Diagnosis at 24 Month Visit
IBIS: Infant Brain Imaging Study
IQ: Intelligence Quotient
LL: Low Familial Likelihood for Autism
LL-: Low Familial Likelihood, No Autism Diagnosis
LORIS: Longitudinal Online Research and Imaging System
MRI: Magnetic Resonance Imaging
TCV: Total Cerebral Volume
