# Supplementary material for: Extra-axial cerebrospinal fluid volumes from 6 to 24 months of age are associated with poorer executive function at school-age in children with and without autism
Source: J Neurodev Disord. 2026 Jan 16;18:9. doi: 10.1186/s11689-025-09671-z (PMC12892752; doi:10.1186/s11689-025-09671-z)
Supplement: Supplementary file 1 — Supplementary Material 1. [file 11689_2025_9671_MOESM1_ESM.docx]

**Supplemental Materials**

**Supplemental Table 1**

**BRIEF Global Executive Composite predicted by EA-CSF and covariates, including EA-CSF and Diagnostic Group Interaction**

| **ANOVA Variable** | ***df (model)*** | ***df (residual)*** | ***F*** | ***p*** |  |  |
| --- | --- | --- | --- | --- | --- | --- |
| Intercept | 1 | 373 | 8554.29 | < .0001 |  |  |
| EA-CSF Volume | 1 | 373 | 22.52 | < .0001 |  |  |
| Total Cerebrum Volume | 1 | 373 | 0.53 | .466 |  |  |
| DAS-II GCA Score | 1 | 373 | 17.43 | < .0001 |  |  |
| Sex | 1 | 373 | 0.02 | .904 |  |  |
| Diagnostic Group | 2 | 4 | 24.51 | .01 |  |  |
| EA-CSF*Group | 2 | 373 | 1.90 | .151 |  |  |
|  |  |  |  |  |  |  |
| **Predictor Variable** | ***df*** | ***β*** | ***SE*** | **95% *CI*** | ***t*** | ***p*** |
| Intercept | 373 | 0.66 | 0.17 | [0.32, 0.99] | 3.86 | < .001 |
| EA-CSF Volume | 373 | 0.26 | 0.09 | [0.09, 0.43] | 3.03 | .003 |
| Total Cerebrum Volume | 373 | 0.03 | 0.05 | [-0.07, 0.13] | 0.64 | .520 |
| DAS-II GCA Score | 373 | -0.20 | 0.05 | [-0.30, -0.11] | -4.13 | < .001 |
| Sex: male | 373 | 0.02 | 0.10 | [-0.18, 0.22] | 0.21 | .835 |
| Diagnostic Group: LL- | 4 | -0.89 | 0.18 | [-1.38, -0.39] | -5.00 | .008 |
| Diagnostic Group: HL- | 4 | -0.71 | 0.17 | [-1.17, -0.25] | -4.30 | .013 |
| EA-CSF*Group LL- | 373 | -0.26 | 0.14 | [-0.52, 0.01] | -1.89 | .06 |
| EA-CSF*Group HL- | 373 | -0.06 | 0.11 | [-0.28, 0.15] | -0.56 | .573 |

Note. Diagnostic Group variables are referenced against HL+. HL+ = high familial likelihood for autism and autism diagnosis; HL- = high familial likelihood for autism and no autism diagnosis; LL- = low familial likelihood for autism and no autism diagnosis. df = degrees of freedom, SE = standard error, CI = confidence interval.

**Supplemental Table 2**

**Conners Executive Function predicted by EA-CSF and covariates, including EA-CSF and Diagnostic Group Interaction**

| **ANOVA Variable** | ***df (model)*** | ***df (residual)*** | ***F*** | ***p*** |  |  |
| --- | --- | --- | --- | --- | --- | --- |
| Intercept | 1 | 373 | 7053.90 | < .0001 |  |  |
| EA-CSF Volume | 1 | 373 | 12.03 | < .001 |  |  |
| Total Cerebrum Volume | 1 | 373 | 0.22 | .639 |  |  |
| DAS-II GCA Score | 1 | 373 | 12.85 | < .001 |  |  |
| Sex | 1 | 373 | 3.70 | .055 |  |  |
| Diagnostic Group | 2 | *4* | 13.40 | .017 |  |  |
| EA-CSF*Group | 2 | 373 | 0.00 | .997 |  |  |
|  |  |  |  |  |  |  |
| **Predictor Variable** | ***df*** | ***β*** | ***SE*** | **95% *CI*** | ***t*** | ***p*** |
| Intercept | 373 | 0.40 | 0.17 | [0.06, 0.74] | 2.32 | .021 |
| EA-CSF Volume | 373 | 0.19 | 0.09 | [0.01, 0.36] | 2.05 | .041 |
| Total Cerebrum Volume | 373 | 0.01 | 0.05 | [-0.09, 0.11] | 0.22 | .826 |
| DAS-II GCA Score | 373 | -0.18 | 0.05 | [-0.28, -0.08] | -3.57 | < .001 |
| Sex: male | 373 | -0.22 | 0.11 | [-0.43, -0.01] | -2.09 | .037 |
| Diagnostic Group: LL- | 4 | -0.54 | 0.18 | [-1.03, -0.06] | -3.10 | .036 |
| Diagnostic Group: HL- | 4 | -0.16 | 0.16 | [-0.61, 0.30] | -0.96 | .392 |
| EA-CSF*Group LL- | 373 | -0.01 | 0.14 | [-0.29, -0.28] | -0.04 | .966 |
| EA-CSF*Group HL- | 373 | -0.01 | 0.11 | [-0.24, -0.22] | -0.08 | .935 |

Note. Diagnostic Group variables are referenced against HL+. HL+ = high familial likelihood for autism and autism diagnosis; HL- = high familial likelihood for autism and no autism diagnosis; LL- = low familial likelihood for autism and no autism diagnosis. df = degrees of freedom, SE = standard error, CI = confidence interval.
